# Short-term response of primary human meniscus cells to simulated microgravity

**DOI:** 10.1186/s12964-024-01684-w

**Published:** 2024-06-21

**Authors:** Zhiyao Ma, David Xinzheyang Li, Xiaoyi Lan, Adam Bubelenyi, Margaret Vyhlidal, Melanie Kunze, Mark Sommerfeldt, Adetola B. Adesida

**Affiliations:** 1 Department of Surgery, Faculty of Medicine and Dentistry, University of Alberta, Edmonton, AB T6G 2R3 Canada; 2 Department of Civil and Environmental Engineering, Faculty of Engineering, AB, University of Alberta, Edmonton, T6G 2R3 Canada; 3 Department of Mechanical Engineering, Faculty of Engineering, University of Alberta, Edmonton, AB T6G 2R3 Canada; 4 Faculty of Science, AB, University of Alberta, Edmonton, T6G 2R3 Canada

**Keywords:** Primary cells, Tissue engineering, Knee osteoarthritis, Sex characteristics, Weightlessness simulation, RNA-seq

## Abstract

**Background:**

Mechanical unloading of the knee articular cartilage results in cartilage matrix atrophy, signifying the osteoarthritic-inductive potential of mechanical unloading. In contrast, mechanical loading stimulates cartilage matrix production. However, little is known about the response of meniscal fibrocartilage, a major mechanical load-bearing tissue of the knee joint, and its functional matrix-forming fibrochondrocytes to mechanical unloading events.

**Methods:**

In this study, primary meniscus fibrochondrocytes isolated from the inner avascular region of human menisci from both male and female donors were seeded into porous collagen scaffolds to generate 3D meniscus models. These models were subjected to both normal gravity and mechanical unloading via simulated microgravity (SMG) for 7 days, with samples collected at various time points during the culture.

**Results:**

RNA sequencing unveiled significant transcriptome changes during the 7-day SMG culture, including the notable upregulation of key osteoarthritis markers such as *COL10A1*, *MMP13*, and *SPP1*, along with pathways related to inflammation and calcification. Crucially, sex-specific variations in transcriptional responses were observed. Meniscus models derived from female donors exhibited heightened cell proliferation activities, with the JUN protein involved in several potentially osteoarthritis-related signaling pathways. In contrast, meniscus models from male donors primarily regulated extracellular matrix components and matrix remodeling enzymes.

**Conclusion:**

These findings advance our understanding of sex disparities in knee osteoarthritis by developing a novel in vitro model using cell-seeded meniscus constructs and simulated microgravity, revealing significant sex-specific molecular mechanisms and therapeutic targets.

**Supplementary Information:**

The online version contains supplementary material available at 10.1186/s12964-024-01684-w.

## Background

Osteoarthritis (OA) is a degenerative disease that commonly affects mechanical load-bearing joints, with the highest prevalence in the knee. The most notable feature of knee osteoarthritis (KOA) is the atrophy of articular cartilage. However, damage and inflammation of tissues that support the knee, such as the meniscus, also contribute to the overall deterioration of the joint [1]. The crucial role of the meniscus in the early onset of KOA was demonstrated by spatial biomechanical mapping of the human knee joint, which revealed that KOA altered the biomechanical properties of menisci before the articular cartilage [2]. Despite the critical impact of the meniscus on the development of KOA, there is a notable gap in research focused on the cellular and molecular mechanisms of meniscus alteration in KOA. While most studies have concentrated on how traumatic meniscus injuries and tears initiate and progress OA in the knee [3–5], few have explored the underlying cellular and molecular dynamics. The prevalence and severity of KOA are disproportionally higher in females [6, 7]. This discrepancy may be attributed to anatomical differences in the knee [7], as well as genetic factors and sex hormones [1, 8]. However, no single factor can fully explain the observed sex differences in KOA. Furthermore, these factors exert their effects by modifying relevant molecules at the cellular level. Hence, it is imperative to understand the molecular and cellular differences to gain insight into the sex differences in KOA.

Research into OA and the differences between sexes is hindered by the shortcomings of existing in vivo and in vitro models [9, 10]. These models struggle to accurately represent the complex and multifaceted nature of OA, especially its initiation and early progression mechanisms. While animal models shed light on OA’s development and possible interventions, discrepancies in biomechanics, molecular pathways, and progression rates limit their applicability to human OA. In vitro studies, though useful for examining specific molecular phenomena, risk oversimplification and often involve cells that lose their original tissue-specific properties, moving away from an accurate portrayal of OA’s biological and pathological dynamics in humans. This issue becomes even more pronounced when exploring sex differences, as variations in responses to OA development and treatments between male and female models mirror the differences seen in human OA prevalence and severity. Consequently, there is an urgent need for the development of more suitable models that can specifically address these sex differences, focusing on cellular signaling discrepancies. Mechanical stressors, including prior knee injuries, muscle weakness, joint misalignment, and obesity, are widely considered significant contributors to the development of KOA [6, 7, 11]. This notion is supported by studies demonstrating that mechanical loading is essential for normal joint function, and prolonged mechanical unloading of joints can lead to degenerative changes resembling KOA [12, 13]. The utilization of real and simulated microgravity (SMG) has become a prominent tool for studying the effects of mechanical unloading on cartilage tissue at both the cellular and tissue levels [14–16]. In several studies, SMG was found to activate the Wnt-signaling pathway [14], leading to increased cartilage catabolism, reduced glycosaminoglycan (GAG) content, increased *MMP3* and *MMP13* expression, and an upregulation of cell apoptosis [17–20]. Although there are fewer investigations examining the effects of short-term microgravity culture compared to long-term culture, several studies using parabolic flight maneuvers demonstrated an acute adaptation of cellular activity to altered mechanical environments [21, 22], but further research is necessary to elucidate the underlying mechanisms that govern the response of primary cells that are devoid of in vitro expansion culture on traditional tissue culture plastic.

Considering the above, this study aims to create an in vitro model via tissue engineering by seeding primary human meniscus fibrochondrocytes from healthy female and male donors into porous collagen scaffolds to generate 3D meniscus models. To preserve the original phenotype of the isolated meniscus fibrochondrocytes, both the cell recovery period and the scaffold pre-culture time were minimized [23]. These models were used to investigate OA-like phenotypes through global transcriptome analysis after 7 days in simulated microgravity (SMG) conditions, and to determine if sex-specific expression patterns related to KOA under prolonged unloading are replicated under brief SMG conditions.

## Methods

### Ethical approval

The experimental protocols and tissue procurement procedures adhered to the ethical guidelines prescribed by the Biomedical Panel of the University of Alberta’s Health Research Ethics Board (Study ID: Pro00018778).

### Plate coating

T75 tissue culture flasks (Sarstedt, Germany) was coated with native human-derived extracellular matrix (ECM) (HumaMatrix Coat, 1 mg/mL, Humabiologics Inc, AZ, USA). Specifically, this matrix was obtained from the human placenta donated by parents who have undergone a minimum of 36 weeks of gestation and whose babies were delivered by Caesarean section. The source materials for the ECM met all relevant industry standards, and the donors were screened and found negative for HIV-1, HIV-2, hepatitis B, hepatitis C, syphilis, and other infectious diseases. The ECM coating solution was prepared by diluting it to a concentration of 0.1 mg/mL in 10–20 mM hydrochloric acid solution with a pH range of 1.9–2.1. Subsequently, 125 µL/cm^2^ of the ECM coating solution was evenly spread on the culture plate surface. The plate was then incubated at 37 °C for 2 hours, excess solution was removed, and the coated surface was left to air-dry overnight without the lid in a cell culture hood. The residual acid was rinsed off the coated surface with sterile phosphate-buffered saline before use.

### Human meniscus fibrochondrocytes (MFC) isolation and plating

MFC were isolated from fresh inner avascular meniscus specimens obtained from 3 male subjects (22, 27, and 32 years old) and 3 female subjects (24, 32, and 38 years old) who had undergone partial meniscectomy by a sports medicine orthopaedic surgeon for acute injury, but with no history of osteoarthritis. The MFC were isolated by 22-hour digestion at 37 °C using type II collagenase (0.15% w/v; 300 U/mg solid; Worthington, NJ, USA) in high glucose DMEM (4.5 mg/mL D-Glucose) supplemented with 5% v/v inactivated FBS (Sigma-Aldrich Co., MO, USA). Isolated primary cells were seeded at a density of 1 × 10^4^ cells/cm^2^ on the ECM-coated plate and cultured in a standard DMEM-complete medium containing high glucose DMEM supplemented with 10% v/v FBS (Sigma-Aldrich), 100 U/mL penicillin, 100 µg/mL streptomycin, 2 mM L-glutamine, and 10 mM HEPES (Sigma-Aldrich) under normal oxygen tension (~ 21% O2) at 37 °C for 48 h.

### Scaffold seeding

After 48 h of culture, adherent primary cells were detached using trypsin-EDTA (0.05% w/v) and seeded onto cylindrical type I collagen scaffolds (diameter = 4 mm, height = 3.5 mm, pore size = 115 ± 20 μm, Integra Lifesciences, USA) [24] at a density of 5 × 10^6^ /cm^3^ [25]. Cells from each donor were used to seed scaffolds and generate cell-seeded meniscus constructs independently. The cell-seeded scaffolds were then pre-cultured for 48 h in serum-free standard chondrogenic medium (high glucose DMEM, HEPES buffer (10 mM), penicillin-streptomycin-glutamine (PSG), dexamethasone (100 nM), ascorbic acid 2-phosphate (365 µg/mL), human serum albumin (125 µg/mL), 40 µg/mL L-proline (all from Sigma-Aldrich), and ITS + 1 premix (Corning, Discovery Labware, Inc., MA, USA)) supplemented with 10 ng/mL TGF-β3 (Proteintech Group, United States, #HZ-1090).

### Mechanical stimulation

After 48 h of pre-culture, the cell-seeded meniscus constructs were randomly allocated to static control group and SMG group. The static control group was cultured in a non-adherent tissue culture well plate, while the SMG group was cultured using a commercially available bioreactor (RCCS-4; Synthecon Inc., TX, USA). The rotation speed of the bioreactor was adjusted to maintain the constructs in a free-falling position, with a speed of 30 rpm applied from day 1 to 2, and 34 rpm from day 3 to 7. During the 7-day experimental period, both the static and SMG groups were cultured in a serum-free chondrogenic medium, supplemented with TGF-β3 (same as pre-culture period), with the volume of culture medium per cell-seeded meniscus construct consistently maintained across both conditions. Samples were collected at day 0 (common control), and from both groups at day 1, day 3, and day 7 (*n* = 2 for each time point).

### RNA extraction and RT-qPCR

The constructs collected at each time point were immediately preserved in Trizol reagent (Life Technologies, United States) and stored at -80 °C until RNA extraction. The PuroSPIN Total RNA Purification KIT (Luna Nanotech, Canada) was used for RNA extraction and purification according to the manufacturer’s instructions. The extracted RNA underwent bulk RNA sequencing and reverse transcription-quantitative polymerase chain reaction (RT-qPCR). For RT-qPCR, a panel of selected chondrogenic (*ACAN, COL1A2, COL2A1*, and *SOX9*), OA-related (*COL10A1, IHH, MMP13*, and *SPP1*), as well as the mechano-transduction (*TRPV4*) markers was examined. The gene expression levels were normalized against the average of three reference genes (*ACTB, B2M*, and *YWHAZ*). The cycle threshold (CT) values of these housekeeping genes across all included donors under both static and SMG conditions is include in supplementary Table 6.

### Bioinformatics

The Biomedical Research Centre at the University of British Columbia used the Illumina NextSeq 500 platform for bulk RNA sequencing, generating 20 million paired end reads with a read length of 42 bp x 42 bp. RNA sequencing data was analyzed using Partek Flow software (Partek Inc, St. Louis, MO, USA). Raw input reads were trimmed and aligned to the reference human genome hg38 using the STAR 2.7.3a aligner. Genes with maximum read counts below 50 were filtered out, and normalization was performed using the Add 1.0, TMM, and Log 2.0 parameters. Statistical analysis was conducted using ANOVA with biological sex and time points as factors. Differentially expressed genes (DEGs) were identified for each comparison by applying *p*-values, adjusted p-values (*q*-values), and fold change (FC) criteria. Partek was used for principal component analysis (PCA), Gene Ontology (GO) and KEGG pathway enrichment analyses, as well as visualization of DEGs using volcano plot and Venn diagrams. Hub protein networks were created for specific groups of DEGs using the Search Tool for the Retrieval of Interacting Genes/Proteins (STRING) online platform. A weighted protein network was constructed for a designated set of DEGs in which all potential protein-protein interactions had a confidence score greater than 0.4. Hub proteins were identified using the Kleinberg hub score metric, and the resulting hub protein network included directly connected proteins. Network visualizations were produced using Cytoscape.

### Statistical analysis

Prism 9 (GraphPad) and Partek Flow software were utilized to conduct the statistical analyses. For RT-qPCR results, statistical analysis employed to compare the expression level across all time points was repeated measures one-way ANOVA with Dunnett’s multiple comparisons test. For RNA sequencing results, student t-test was employed to compare the expression level of two selected time points or between male and female groups. Multiple test correction FDR step-up (*q*-value) was performed on the *p*-values of each comparison.

## Results

### Dataset overview

**Fig. 1:**
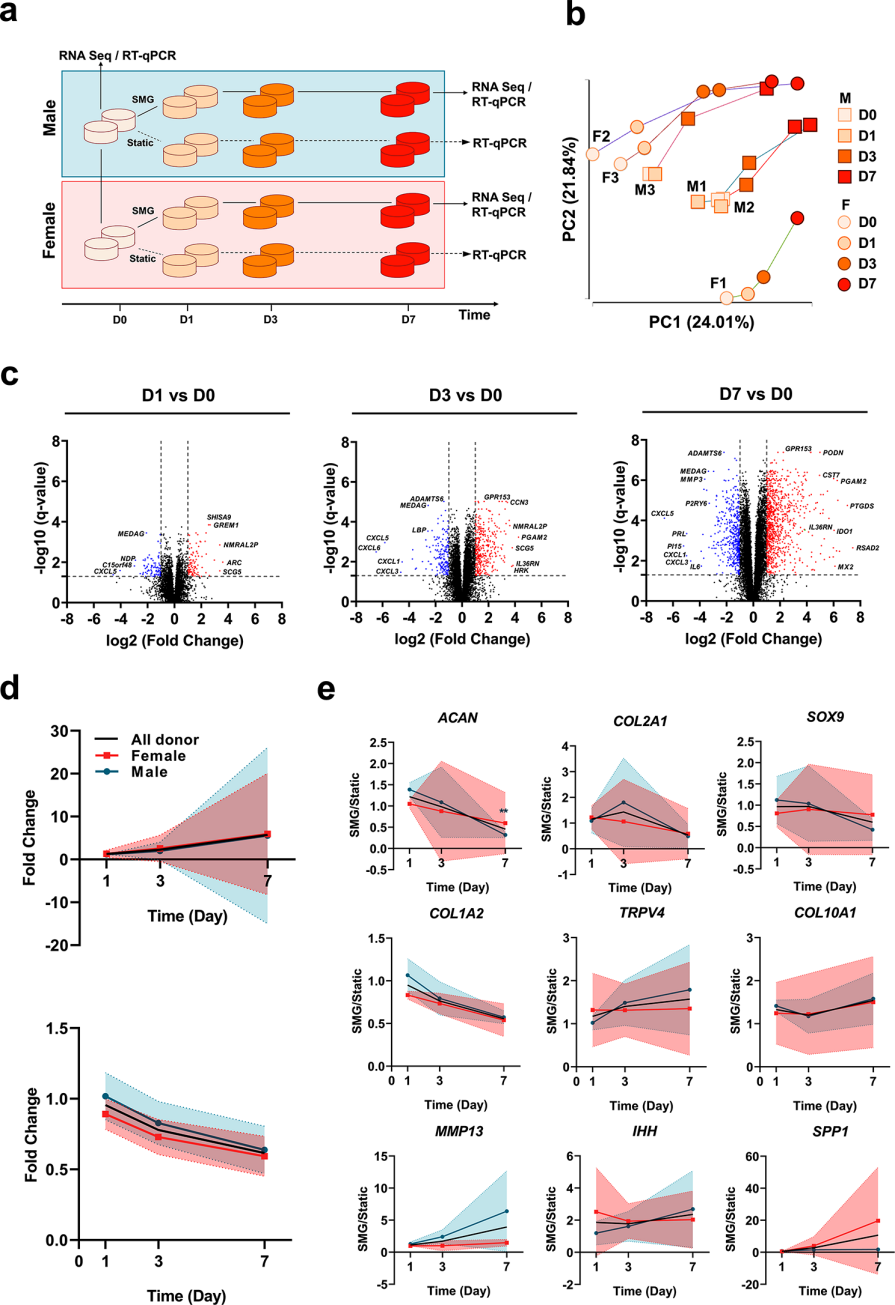
RNA sequencing and RT-qPCR-based transcriptome across all donors. **a** Schematic for analyzing temporal trajectories of cell-seeded meniscus constructs, initiated from a static control. Samples were collected on day 0 under static control, as well as on day 1, 3, and 7 under SMG condition for RNA-seq analysis. For RT-qPCR analysis, samples were collected on day 0 under static control and on day 1, 3, and 7 for both SMG and static conditions. **b.** Principal component analysis (PCA) of temporal transcriptome trajectory for all donors (*n* = 6). The first two principal components (PC1 and PC2) were plotted, with male and female donors represented by different shapes (square for male and circle for female), and different time points represented by different colors. **c.** Volcano plot of the whole transcriptome on day 1, 3, and 7 compared to day 0 for all donors. Upregulated DEGs (fold change ≥ 2, *q*-value ≤ 0.05) were labelled with red color and downregulated DEGs (fold change ≤ -2, *q*-value ≤ 0.05) were labelled with blue color. **d.** Characterization of the temporal expression trajectory of genes significantly correlated with SMG culture time (absolute partial correlation coefficient ≥ 0.8, *q*-value ≤ 0.05). To assess the temporal correlation, the expression level of each gene in the SMG group was compared at each time point with the expression level of the same gene on day 1 in the static group (fold change). The fold change in expression level was then compared to the trend of culture time to determine the temporal correlation of each gene. Top: positively correlated genes. Bottom: negatively correlated genes. Shaded area: Standard Deviation (SD) **e.** Characterization of the temporal expression trajectory of selected chondrogenic/OA-related signature genes. Data for females, males, and all donors combined were presented as mean values along with their corresponding standard deviations. Shaded area: Standard Deviation (SD).** *p* ≤ 0.005 compared to day 1

In this study, RNA sequencing and RT-qPCR were used to analyze gene expression changes over a short-term culture in SMG with 6 donors (3 males and 3 females) and 4 time points per donor (Fig. 1a). The RNA-sequencing preprocessing pipeline retained 13,537 genes for downstream analysis. The quality assessments of extracted RNA were conducted before sequencing and after each step of preprocessing (Supplementary Table 1). The authenticity of the RNA sequencing data was verified by comparing it to RT-qPCR data, showing a high correlation (R^2^ value of 0.9588) between the two transcriptomic techniques (Supplementary Fig. 1).

### Analysis of transcriptomic trajectory of meniscus constructs

In order to evaluate the alteration of transcriptome profile over time, PCA was employed to examine the temporal changes in the transcriptome profiles across all donors during SMG culture. PC1 and PC2 captured a temporal trajectory, with PC1 (24.01%) accounting for slightly more variation than PC2 (21.84%) (Fig. 1b). SMG culture duration had little impact on gene expression until day 3, and by day 7, donors tended to converge towards a similar transcriptional pattern. Key contributors to the change in transcriptome trajectory were identified (Supplementary Table 2), including genes associated with inflammatory processes (*IL21R*, *ILF2*, and *TNFRSF11B*) and bone development (*BMP6* and *BMPR2*). DEG analysis was performed to identify genes that showed significant statistical differences (|FC| ≥ 2 and *q*-value < 0.05) at each time point (Fig. 1c). Comparison of the volcano plots over time revealed that the impact of SMG became more evident as the number of DEGs, and the extent of regulation considerably increased. The expression of several genes involved in early inflammatory processes (*CXCL* families), the NF-κB signaling pathway (*IL36RN*), and the matrix remodeling process (*ADAMTS6*) were consistently and significantly regulated during the SMG culture period (Fig. 1c).

To further assess the impact of SMG culture duration on transcriptome profile alteration, the strength of correlation between the expression level changes of each gene and SMG culture time was determined. Among 13,537 genes, 5,064 genes showed a significant correlation with SMG culture duration (*q*-value ≤ 0.05), with 570 genes exhibiting a strong correlation (|partial correlation coefficient| ≥ 0.8). Of these strongly correlated genes, 386 were positively correlated with SMG culture time while 184 were negatively correlated (Fig. 1d) (Supplementary Table 3). The KEGG pathways (Supplementary Table 3) that were enriched by the group of genes that showed strong positive correlations include the glycosaminoglycan degradation pathway (*HEXA* and *HGSNAT*), the rheumatoid arthritis pathway (*CTSK* and *ATP6V* family), inflammation-related pathways (*GSK3B, NCK2, HRH1*, and *ITPR1*), Wnt signaling pathways (*APCDD1L, FZD1, GPC4, GSK3B*, and *MMP7*), hedgehog signaling pathway (*EVC* and *GSK3B*), and PPAR signaling pathways (*ACSL1, FABP4*, and *PLIN2*).

### Examination of early KOA onset mechanisms in the SMG model

**Fig. 2:**
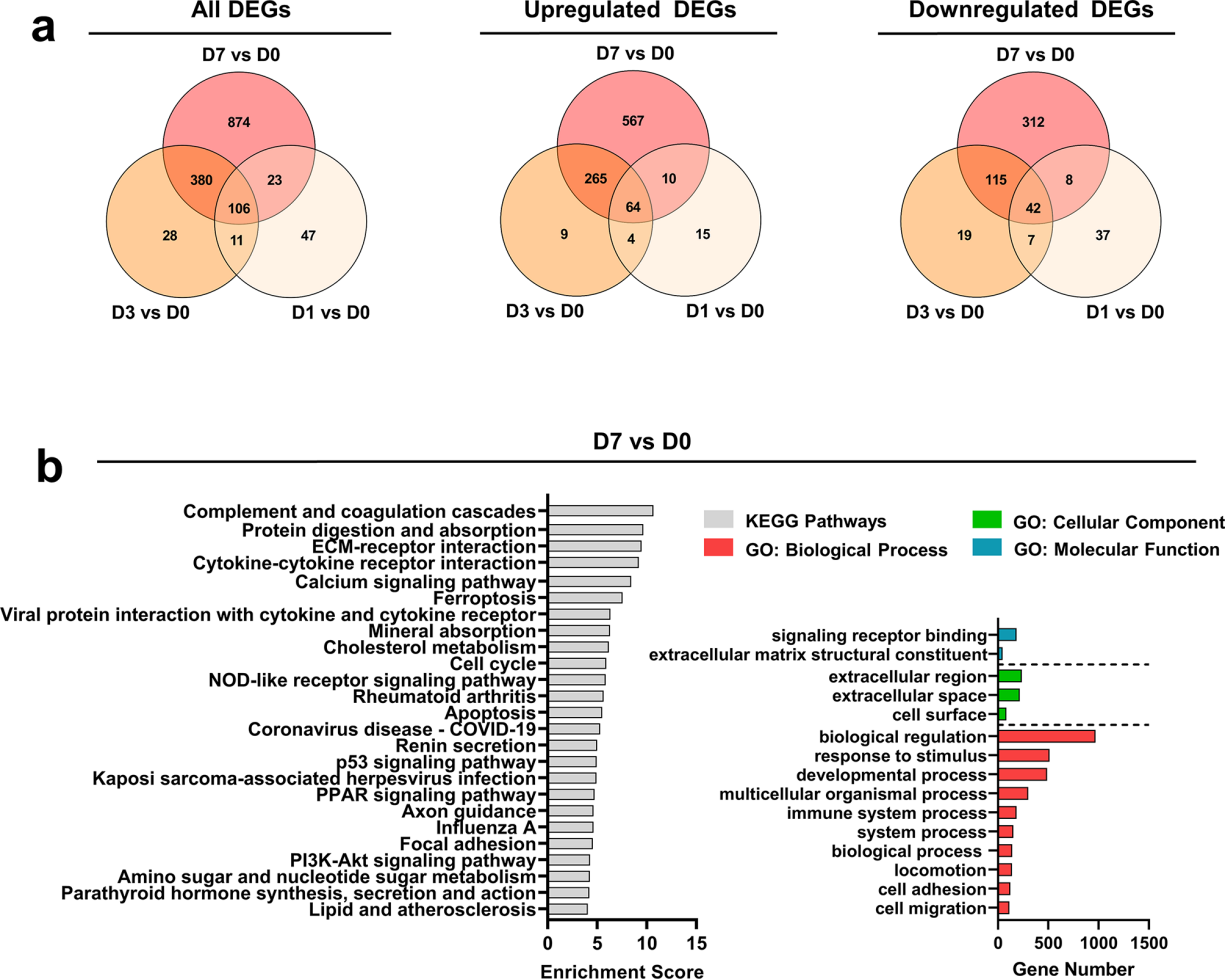
Functional enrichment of altered transcriptome induced by SMG across all donors. **(a)** Venn diagram of the comparison between all DEGs, upregulated DEGs, and downregulated DEGs in all donors (*n* = 6) across three time points. Each circle represents a distinct time point with the number of uniquely regulated genes indicated in the non-overlapping areas, while overlapping areas show the number of genes that are commonly regulated across multiple time points. **(b)** Top non-redundant Gene Ontology (GO) terms and KEGG pathways enriched by all DEGs on day 7

Changes in chondrogenesis and OA-like phenotype were first confirmed using a panel of selected markers by RT-qPCR (Fig. 1e). Chondrogenic markers (*ACAN, COL1A2, COL2A1*, and *SOX9*) initially comparable between groups decreased over time in the SMG culture, while OA-related markers (*IHH, MMP13, SPP1*, and *COL10A1*) increased. *TRPV4* was activated and upregulated in the SMG group, indicating mechanical stimulation responsiveness. Due to large donor variability, only the expression level of *ACAN* was statistically significant at day 7; however, the general trend across all donors aligned with this pattern. DEGs from RNA sequencing were then compared between time points (Fig. 2a), showing a substantial increase in the number of DEGs from day 1 to day 7. Some DEGs were consistently regulated throughout the culture period, suggesting that SMG consistently regulated a specific group of genes with an expanding effect over time.

Functional enrichment analysis was performed to explore the KEGG pathways and GO terms significantly enriched by all DEGs at each time point (Fig. 2b, Supplementary Fig. 2). The findings revealed a consistent overrepresentation of several GO terms, including “extracellular space and regions”, “response to stimulus”, and “signaling receptor binding”. At later time points, additional functional terms such as matrix remodeling and immune system pathways were identified. For KEGG pathway analysis, significant enrichment was observed in pathways such as “calcium signaling pathway,” “complement and coagulation cascades,” “PPAR signaling pathway,” and “rheumatoid arthritis,” which could potentially play a crucial role in the early onset of KOA (Fig. 2b).

To elucidate the underlying molecular mechanism responsible for the OA-inducing effect of SMG, the hub protein networks were constructed for all DEGs at each time point (Supplementary Figs. 3–5). The networks comprised 27, 86, and 111 genes on days 1, 3, and 7, respectively (Supplementary Table 4), with CCL2, IL6, and CDK1 serving as the hub proteins. CCL2 and IL6 are known to participate in immunoregulatory and inflammatory processes, while CDK1 plays a critical role in cell cycle regulation. Notably, several proteases including the MMP family and ADAMTS5 were highly connected to the hub protein IL6 in the network (Supplementary Fig. 4). More than half of the top 25 KEGG pathways enriched by the panel of genes corresponding to the filtered protein network overlapped on day 1 and 3, including several pro-inflammatory signaling pathways and OA-related pathways, such as “IL-17 signaling pathway,” “TNF signaling pathway,” and “rheumatoid arthritis” (Supplementary Table 4).

### Examination of sex-dependent responses to SMG

After establishing SMG’s capability to generate an OA-like phenotype in cell-seeded meniscus construct models, the donors were stratified by sex. The models derived from male and female primary MFC were analyzed separately to investigate sex-specific responses to SMG and identify molecular mechanisms that may contribute to the disproportionate incidence and severity of OA in females.

**Fig. 3:**
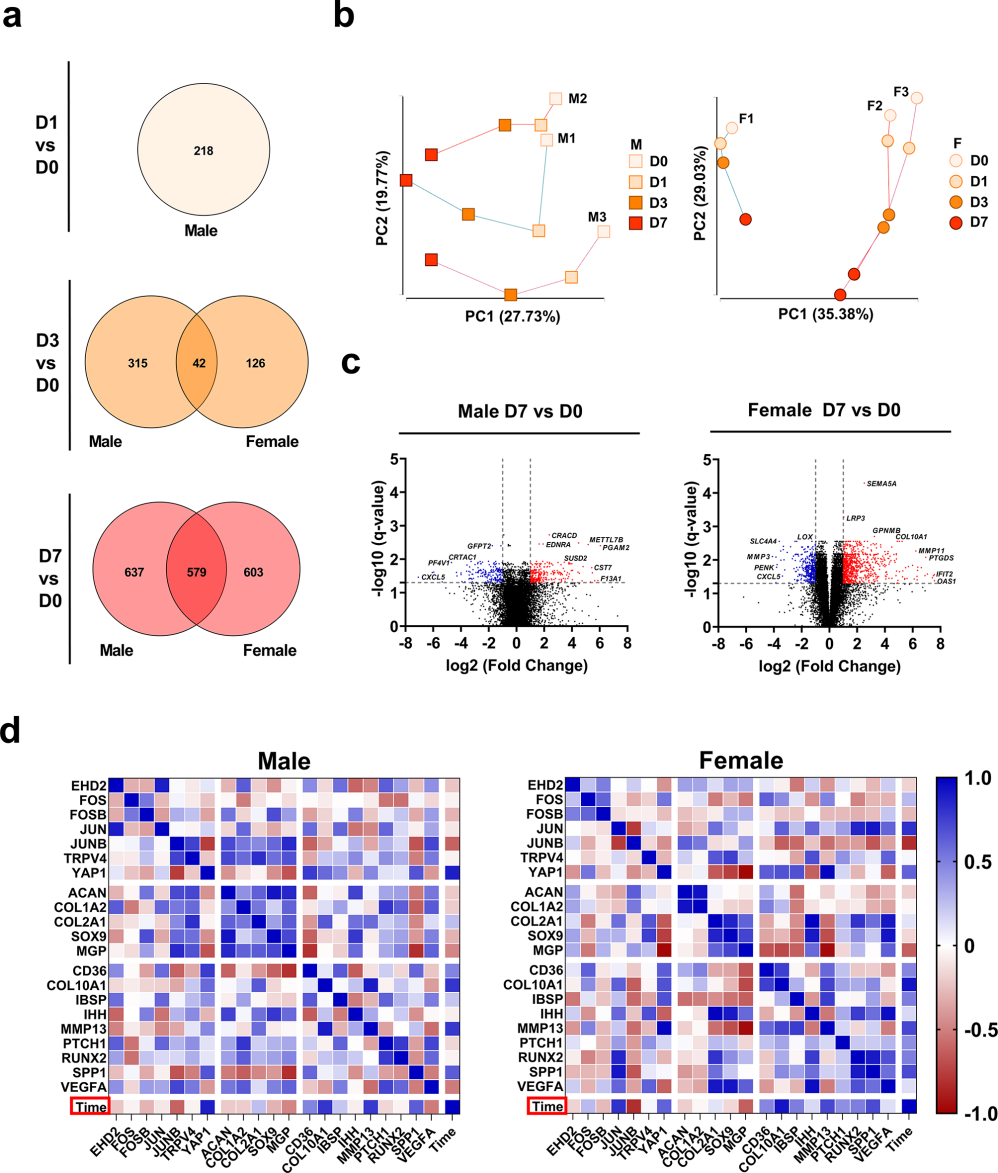
RNA sequencing based transcriptome profiling and trajectory analysis for male and female donors. **(a)** Venn diagram of the comparison between all DEGs regulated in male (*n* = 3) and female (*n* = 3) donors across three time points. **(b)** Principal component analysis (PCA) of temporal transcriptome trajectory for male and female donors. The first two principal components (PC1 and PC2) were plotted. **(c)** Volcano plot of the whole transcriptome on day 1, 3, and 7 compared to day 0 for male and female donors. Upregulated DEGs (fold change ≥ 2, *q*-value ≤ 0.05) were labelled with red color and downregulated DEGs (old change ≤ -2, *q*-value ≤ 0.05) were labelled with blue color. **(d)** Pearson correlation heatmap of select markers and culture time for male and female donors. Heatmap was generated by calculating the pairwise Pearson correlation coefficient, with the color of each cell representing the corresponding coefficient value

DEGs were identified for male and female donors at each time point. SMG regulated 1,182 genes in female donors on day 7, while the number of DEGs increased gradually from 218 genes on day 1 to 1,216 genes on day 7 for male donors. Only a small proportion of DEGs were shared on day 3, while approximately half of the DEGs were the same on day 7 when comparing male and female donors at the same time points (Fig. 3a). This trend was also observed when the up and downregulated DEGs were separated (Supplementary Fig. 6). The results of the PCA analysis revealed a noticeable impact of SMG on the transcriptome profile over culture time for both male and female donors (Fig. 3b). Key genes associated with OA development were among the most significantly regulated DEGs in female donors on day 7, such as *LOX* (-5.13-fold), *MMP3* (-17.37-fold), *MMP11* (74.71-fold), and *COL10A1* (29.53-fold) (Fig. 3c).

Correlation analysis was conducted to investigate the relationship between well-established cartilage and OA-related markers within male and female donor groups. Correlation heatmaps were created using selected signature markers divided into mechano-transduction (*EHD2, FOS, FOSB, JUN, JUNB, TRPV4*, and *YAP1*), chondrogenesis (*ACAN, COL1A2, COL2A, SOX9*, and *MGP*), and OA development (*CD36, COL10A1, IBSP, IHH, MMP13, PTCH1, RUNX2, SPP1*, and *VEGFA*) subcategories (Fig. 3d). The expression levels of chondrogenic markers showed a negative correlation with SMG culture time in both male and female donors, while the correlation with OA markers was positive, especially for females. The correlation patterns of OA markers and chondrogenic markers differed for males and females, particularly with regards to *ACAN* and *COL1A2*, which exhibited opposing trends. These suggested the potential unique interaction mechanism between these markers within each sex group. Finally, among the chosen mechano-transduction markers, *JUN* and *JUNB* exhibited the most notable sex-specific differences in their correlation patterns with other markers.

**Fig. 4:**
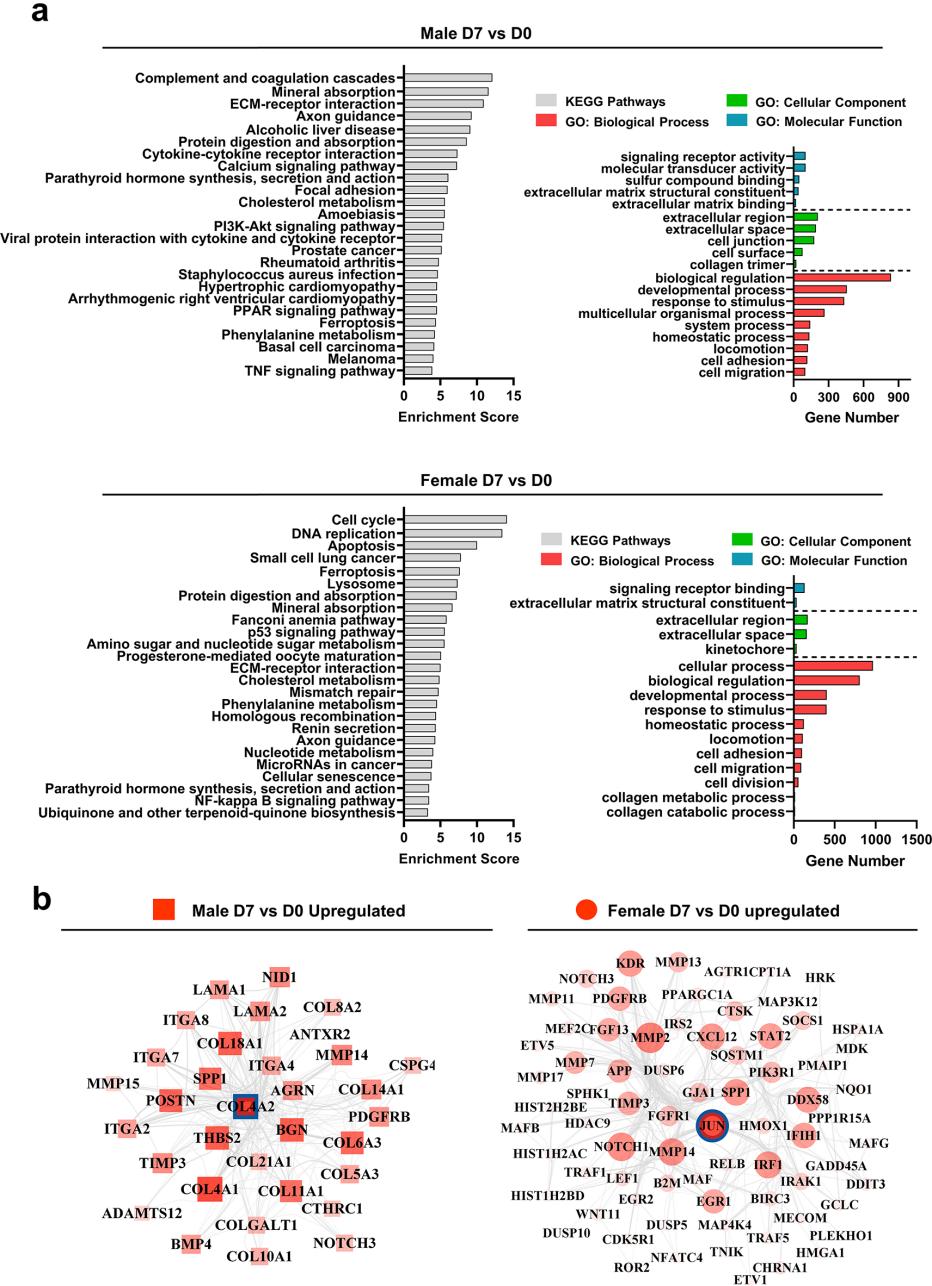
Functional enrichment of altered transcriptome induced by SMG for male and female donors. **(a)** Top non-redundant Gene Ontology (GO) terms and KEGG pathways enriched by all DEGs in male and female donors on day 7. **(b)** Hub protein networks for upregulated DEGs on day 7 in male and female donors. Hub protein was defined as the protein with the highest connectivity. A network was generated by including the hub protein and its direct connections with all other proteins. The size and density of color of each protein in the network reflected its level of connectivity, with larger and denser colors indicating higher connectivity. The hub protein in the net work was highlighted with a blue circle. The hub protein for male donors was identified as COL4A2, and for female donors was JUN

Functional enrichment analysis showed that both male and female donors demonstrated comparable GO enrichment on day 3 and day 7, with general biological processes such as cell adhesion and migration, extracellular matrix components, and molecular function of signaling receptor binding being the primary terms. The KEGG pathway “mineral absorption” was enriched in both males and females on day 3 and day 7, along with several pathways related to the cell cycle and apoptosis. Inflammation and immune response were significant functional enrichments for male donors on both day 3 and day 7, but not for female donors (Fig. 4a, Supplementary Fig. 7).

To investigate further the molecular mechanism underlying sex-dependent differences, hub gene networks were constructed for male and female donors separately, considering all DEGs combined, up-regulated DEGs, and down-regulated DEGs (Fig. 4b, Supplementary Fig. 8). Upon comparing males and females, it was found that the corresponding hub protein from up-regulated DEGs on both day 3 and day 7 in females were primarily associated with Wnt signaling (CTSK, IRAK1, JUN, PIK3R1, LEF1, MMP7, NFATC4, ROR2, and WNT11), VEGF signaling (KDR, PIK3R1, and SPHK1), and NF-κB signaling pathways (BIRC3, CXCL12, DDX58, GADD45A, IRAK1, RELB, TRAF1, and TRAF5). In contrast, the hub components of upregulation in males were ECM components and matrix remodeling enzymes (Supplementary Table 5).

## Discussion

The absence of adequate model systems for studying the molecular pathogenesis of KOA impedes our comprehension of the entire disease spectrum. Studies that use end-stage disease samples may not capture essential molecular changes that occur earlier in the disease progression, leading to an incomplete understanding of the underlying mechanisms. By employing tissue engineering techniques and coating cell contact surfaces with human-derived extracellular components, this model maintains meniscus fibrochondrocytes (MFC) in a state closely resembling their native environment. The meniscus models engineered with primary cells were analyzed by RNA sequencing to establish a transcriptome profile over time and examine the initial changes in molecular profile that contribute to the early onset of KOA.

The molecular mechanisms underlying the connection between meniscus injury and the onset and progression of KOA are intricate and not completely understood. However, it is hypothesized that meniscus injury initiates a series of cellular and molecular reactions that culminate in an inflammatory response within the knee joint, which contributes to the development of KOA [26]. In our study, we used cell-seeded meniscus constructs to demonstrate that molecular patterns and pathways related to inflammation and immune response were significantly enriched at nearly all time points in the SMG treatment. The inflammatory response involves various immune cells, growth factors, cytokines, and chemokines, and prior research has suggested that inflammatory-related molecules primarily drive the enzymatic cascade of OA, which leads to cartilage matrix degradation [27, 28]. After knee injury, IL6 and TNF-α levels rise within 24 h [29] and can persist for up to 18 months post-injury [30]. They regulate various signaling pathways and promote the recruitment and activation of immune cells such as macrophages [31]. CCL2 is another signaling molecule induced after joint injury, primarily recruiting monocytes, and modulating downstream immune response cascades [32]. In our study, the short-term SMG model effectively captured the initial inflammatory events associated with joint injury and early onset KOA observed in various in vitro and in vivo models, with CCL2 and IL6 being the hub protein on day 1 and day 3, respectively. This finding aligns with the observation that regulating these two molecules are critical in modulating the inflammatory response during the acute phase of joint injury, and the onset of KOA. In addition, the functional enrichment analysis showed that TNF and MAPK signaling pathways, IL-17 signaling pathway, as well as complement and coagulation cascades were among the most significantly enriched pathways on both day 1 and 3. Although the precise process behind inflammatory-induced cartilage catabolic activity remains unclear, several studies suggest that inflammatory cytokines play a crucial role in regulating various catabolic enzymes, including proteases from the MMP and ADAMTS families [33, 34]. Our study also demonstrated this correlation, as evidenced by the hub protein network on day 3 for all donors. Several MMPs and ADAMTS5 were closely linked to the hub protein IL6 and other proteins in the network.

In addition to the activation of the inflammation cascade and the degradation of ECM components, another important event that marks the early stage of KOA is the proliferation of chondrocytes [35, 36]. In normal articular cartilage, chondrocytes usually do not undergo proliferation or terminal differentiation. However, in diseased cartilage, there is an augmentation in chondrocyte proliferation and hypertrophic differentiation [37], along with the start of vascularization and focal calcification. Cyclin-dependent kinases (CDKs) are a family of proteins that play a key role in regulating the cell cycle, with CDK1 being the first CDK gene to be identified and conserved across all organisms [38]. The importance of CDK1 in skeletal system development has been demonstrated through loss-of-function experiments on chondrocytes in mouse models [39]. In the context of disease state, CDK1 has been identified as a hub gene in several studies [40, 41]. The findings of our study support that CDK1 plays a crucial role in the early onset of KOA. CDK1 was identified as the hub protein for all differentially expressed genes on day 7 when all donors were combined. Notably, the impact of CDK1 was more pronounced in female donors than in male donors. Specifically, CDK1 was the hub protein exclusively for female donors on day 7, while IL6 was the hub protein for male donors. In addition, the top enriched KEGG pathways demonstrated significant cell proliferation-related activities such as “cell cycle” and “DNA replication” that were only present in female donors.

Cartilage development and maintenance rely on mechanical stimulation. Chondrocytes sense environmental stimuli via the pericellular matrix [42] and convert them into biological signals through various receptors on their cell membranes, known as mechanotransduction [43]. In healthy joints, this process is crucial for preserving cartilage tissue’s integrity and function. However, in OA joints, an imbalance between the mechanical loading and the ability of chondrocytes to respond to mechanical signals can lead to cartilage breakdown and joint degeneration [12, 44]. Chondrocyte mechanotransduction involves various signaling molecules and pathways. Our study assessed the expression of these molecules and compared the expression patterns between males and females. The results showed that *JUN* exhibited the most significant difference in expression correlations with other assessed factors between male and female donors. The JUN proteins are part of the AP-1 transcription factor family and was identified as a regulator of gene expression and a trigger for downstream signaling cascades in response to mechanical stimuli in chondrocytes [45]. The hub protein network analysis in this study demonstrated a sex-dependence of JUN’s expression, where JUN was identified as the hub protein only in female donors. Moreover, the network showed that several proteins from the MMP family and the calcification-related protein SPP1 were highly connected to JUN, suggesting the potential role of JUN in regulating the expression of genes involved in the matrix degradation and calcification processes.

In addition to its role in mechanotransduction, JUN can also crosstalk with other well-established signaling pathways involved in OA. For example, JUN participates in Wnt signaling pathways, which regulate the expression of genes involved in cell proliferation, differentiation, and survival [46, 47]. Numerous studies have shown that, in the context of KOA, there is an upsurge in Wnt signaling that can result in chondrocyte hypertrophy, inflammation, and cartilage deterioration [48, 49]. In this study, the hub protein network of upregulated DEGs for males and females was analyzed, along with the enriched pathways corresponding to the genes in the network. For females with JUN as the hub protein, Wnt signaling pathways were among the top enriched KEGG pathways, along with several other OA-relevant pathways such as VEGF signaling, and NF-kappa B signaling pathways. On the other hand, for males, the hub components of upregulations were ECM components and matrix remodeling enzymes.

One limitation of our study involves using day 0 baseline samples as a common control instead of matched static samples from each time point for RNA-sequencing data analysis. Using matched time-point controls in static conditions could potentially enhance the precision of our findings by more distinctly isolating the effects of simulated microgravity. Another limitation of this study lies in the need for validation experiments to link the transcriptional data with functional outcomes, which is crucial for confirming the model’s predictive power for OA progression and sex differences. Despite this, the study’s innovative use of a refined in vitro model and SMG conditions successfully narrows down potential targets for early KOA development and sex disparities, marking a significant step forward in the search for effective treatments. This approach solidifies the model’s role as a valuable screening platform for identifying promising molecular targets.

## Conclusion

In conclusion, our study opens the perspective that short-term SMG induced molecular events mimicking early KOA in refined human meniscal models developed from non-osteoarthritic primary MFC that can be leveraged for therapeutic discovery and development. Transcriptomic analysis revealed a significant enrichment of genes and pathways related to inflammation and immune response in the early onset of KOA. Notably, our study suggests that sex dimorphism in cellular proliferation and JUN expression may be the key factors responsible for the progression of early-onset KOA.

### Electronic supplementary material

Below is the link to the electronic supplementary material.

Supplementary Figure 1

Supplementary Figure 2

Supplementary Figure 3

Supplementary Figure 4

Supplementary Figure 5

Supplementary Figure 6

Supplementary Figure 7

Supplementary Figure 8

Supplementary Table 1

Supplementary Table 2

Supplementary Table 3

Supplementary Table 4

Supplementary Table 5

Supplementary Table 6

## Data Availability

RNA-Seq data was deposited into the Gene Omnibus Database (GEO) under accession number GSE231848.

## Abbreviations

OA: Osteoarthritis
KOA: Knee osteoarthriti
MFC: Meniscus fibrochondrocytes
SMG: Simulated microgravity
ECM: Extracellular matrix
DEGs: Differentially expressed genes
