# Supplementary figures and images for: Short-term response of primary human meniscus cells to simulated microgravity
Source: Cell Commun Signal. 2024 Jun 21;22:342. doi: 10.1186/s12964-024-01684-w (PMC11191296; doi:10.1186/s12964-024-01684-w)

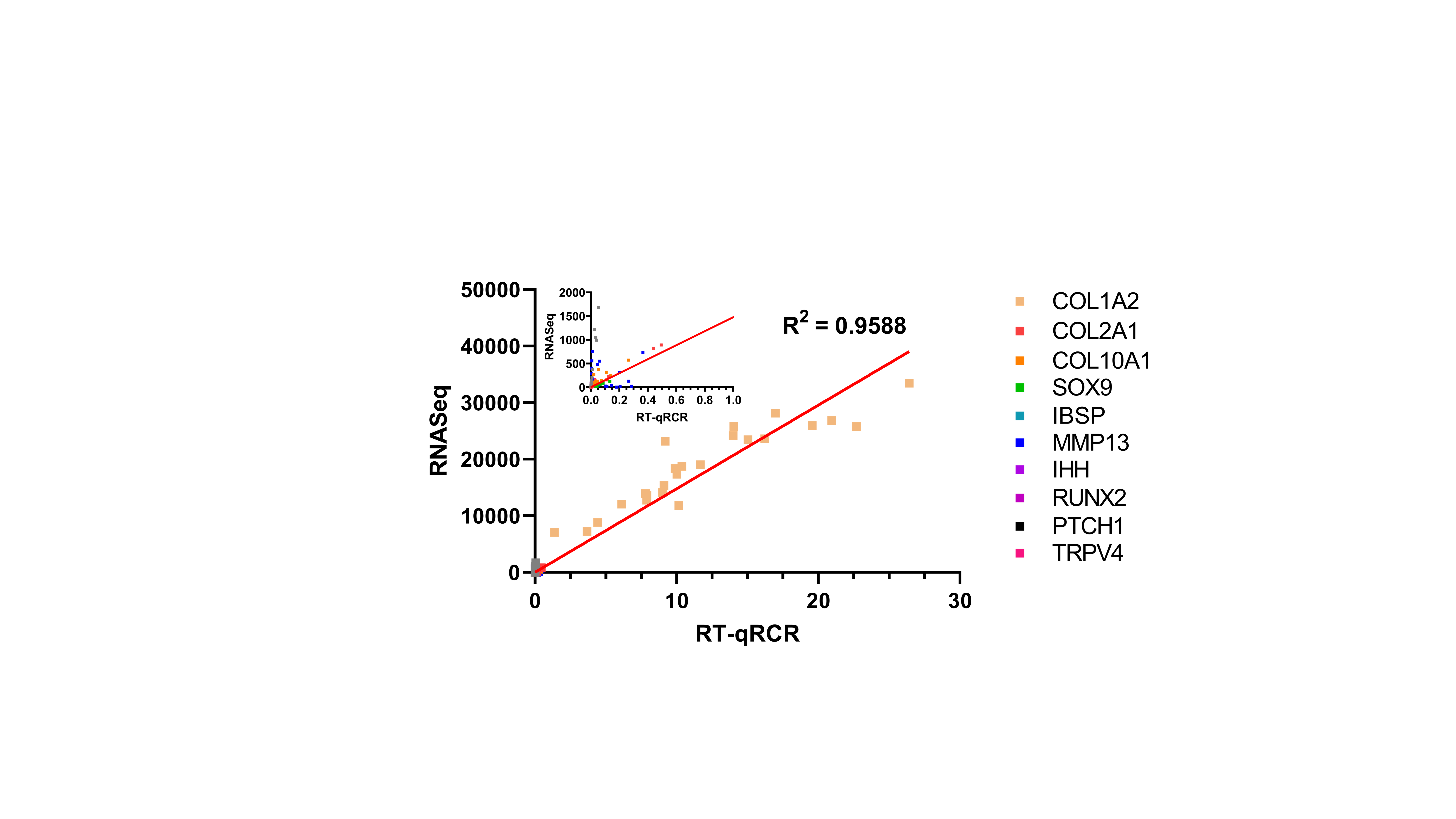

Supplement: Supplementary file 1 — Supplementary Figure 1 [file 12964_2024_1684_MOESM1_ESM.tif]

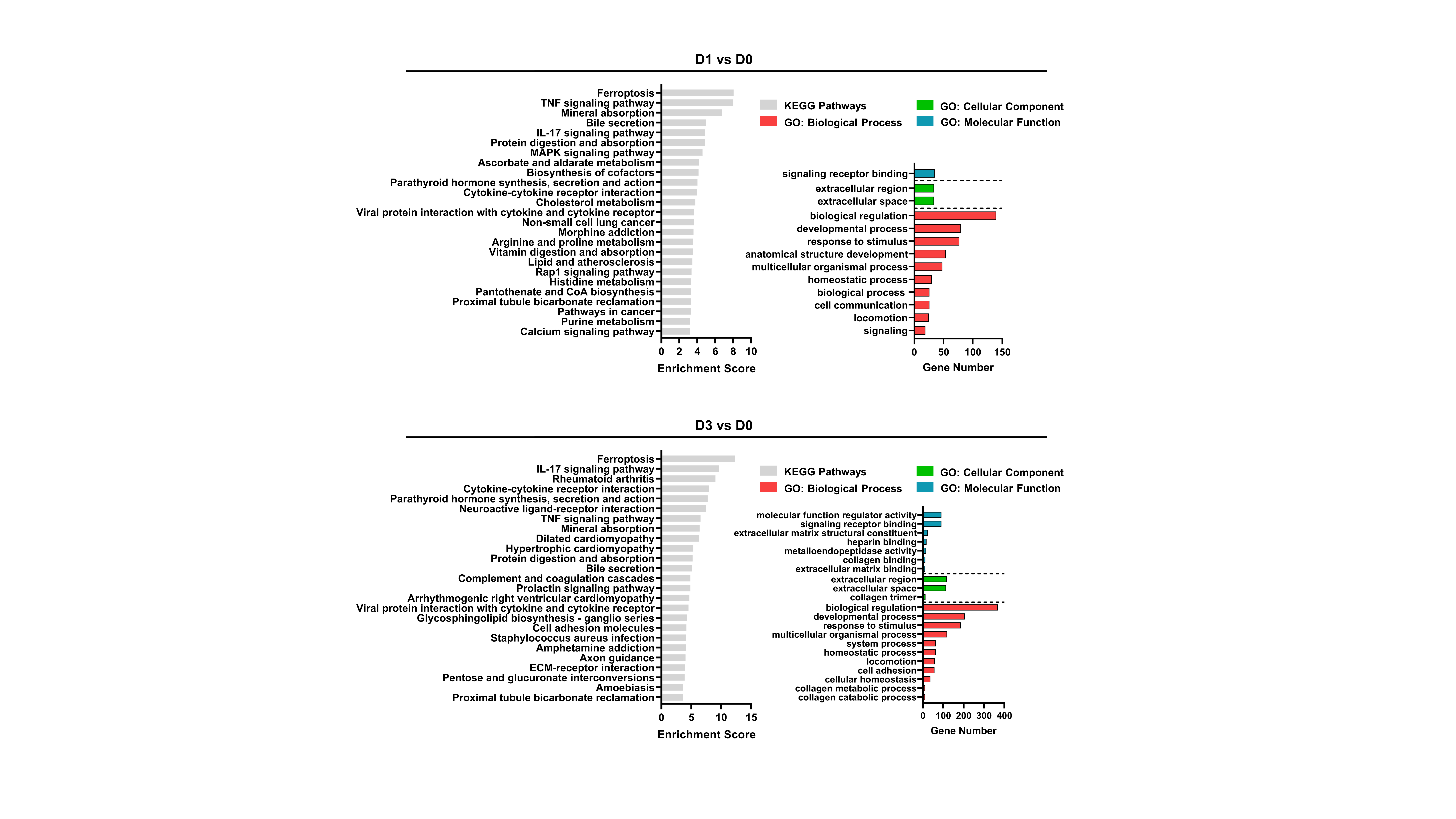

Supplement: Supplementary file 2 — Supplementary Figure 2 [file 12964_2024_1684_MOESM2_ESM.tif]

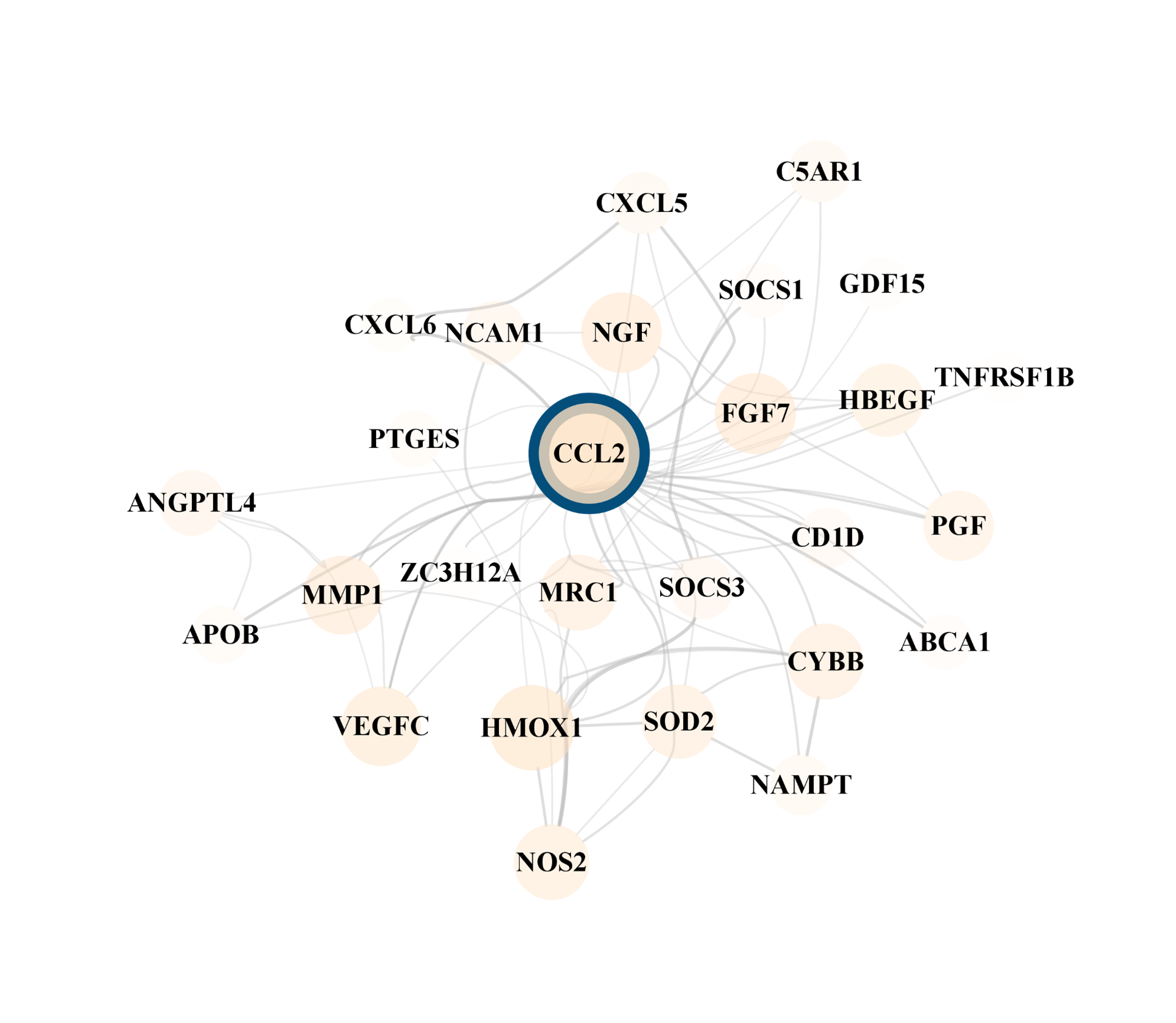

Supplement: Supplementary file 3 — Supplementary Figure 3 [file 12964_2024_1684_MOESM3_ESM.tif]

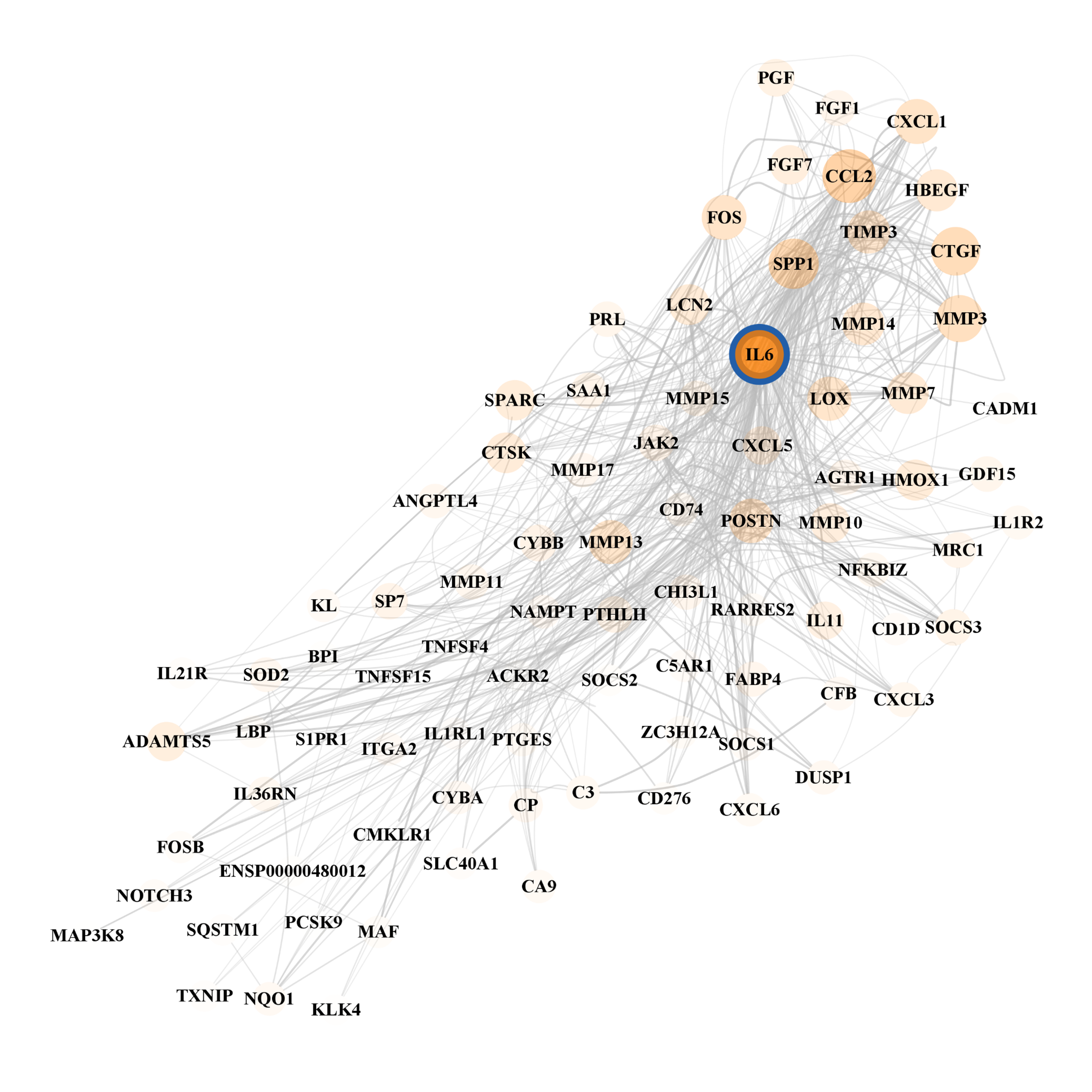

Supplement: Supplementary file 4 — Supplementary Figure 4 [file 12964_2024_1684_MOESM4_ESM.tif]

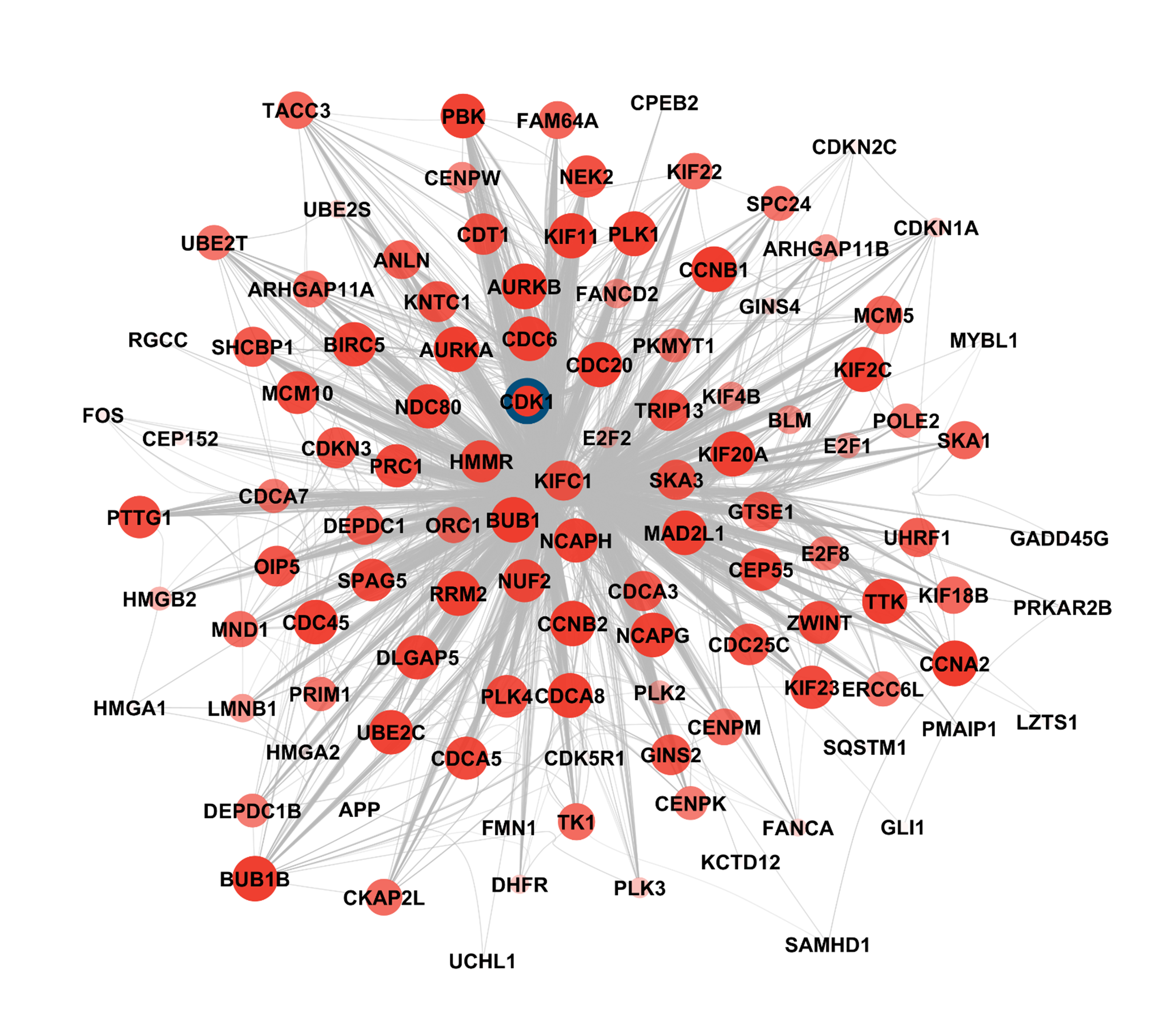

Supplement: Supplementary file 5 — Supplementary Figure 5 [file 12964_2024_1684_MOESM5_ESM.tif]

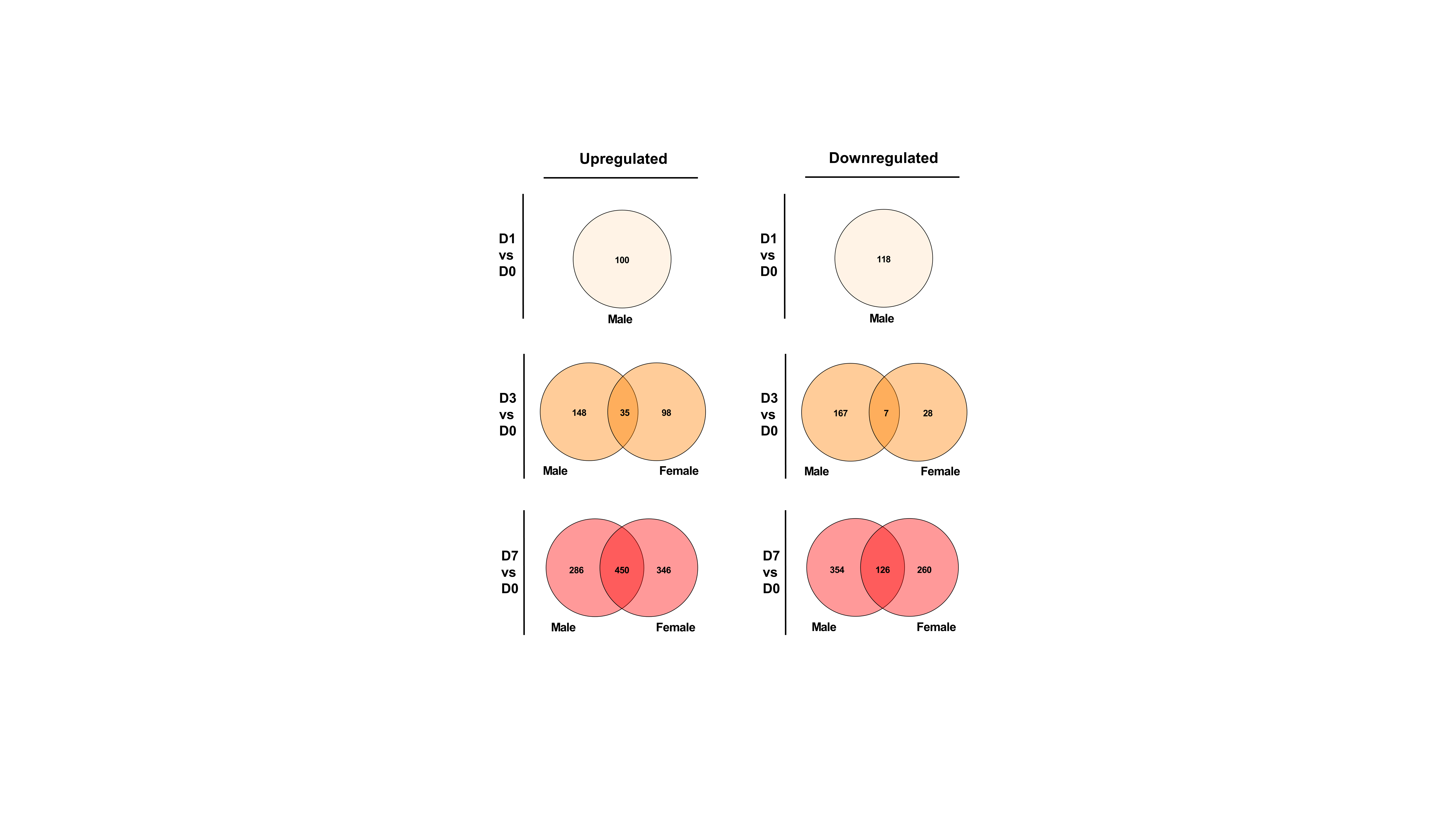

Supplement: Supplementary file 6 — Supplementary Figure 6 [file 12964_2024_1684_MOESM6_ESM.tif]

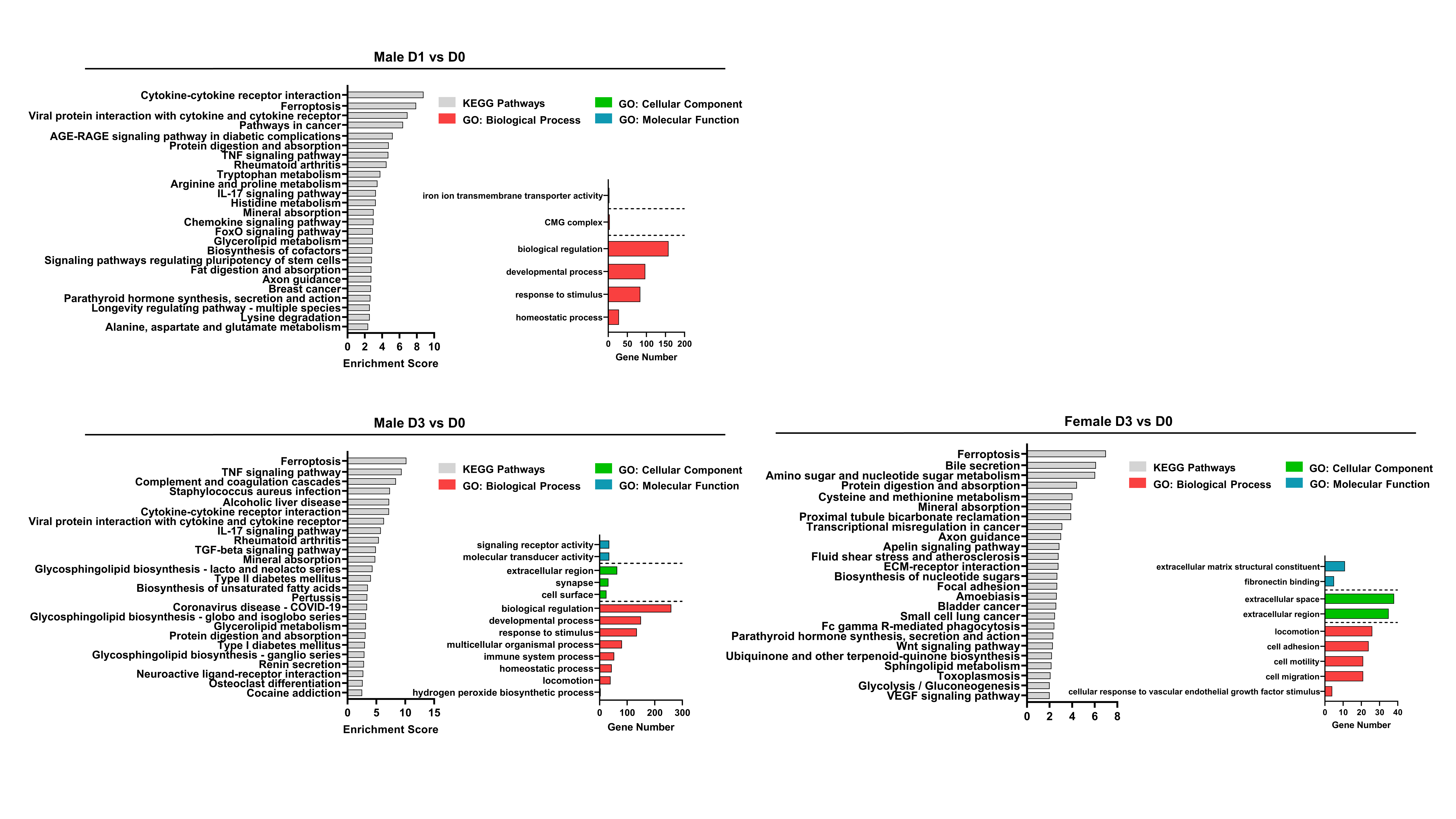

Supplement: Supplementary file 7 — Supplementary Figure 7 [file 12964_2024_1684_MOESM7_ESM.tif]

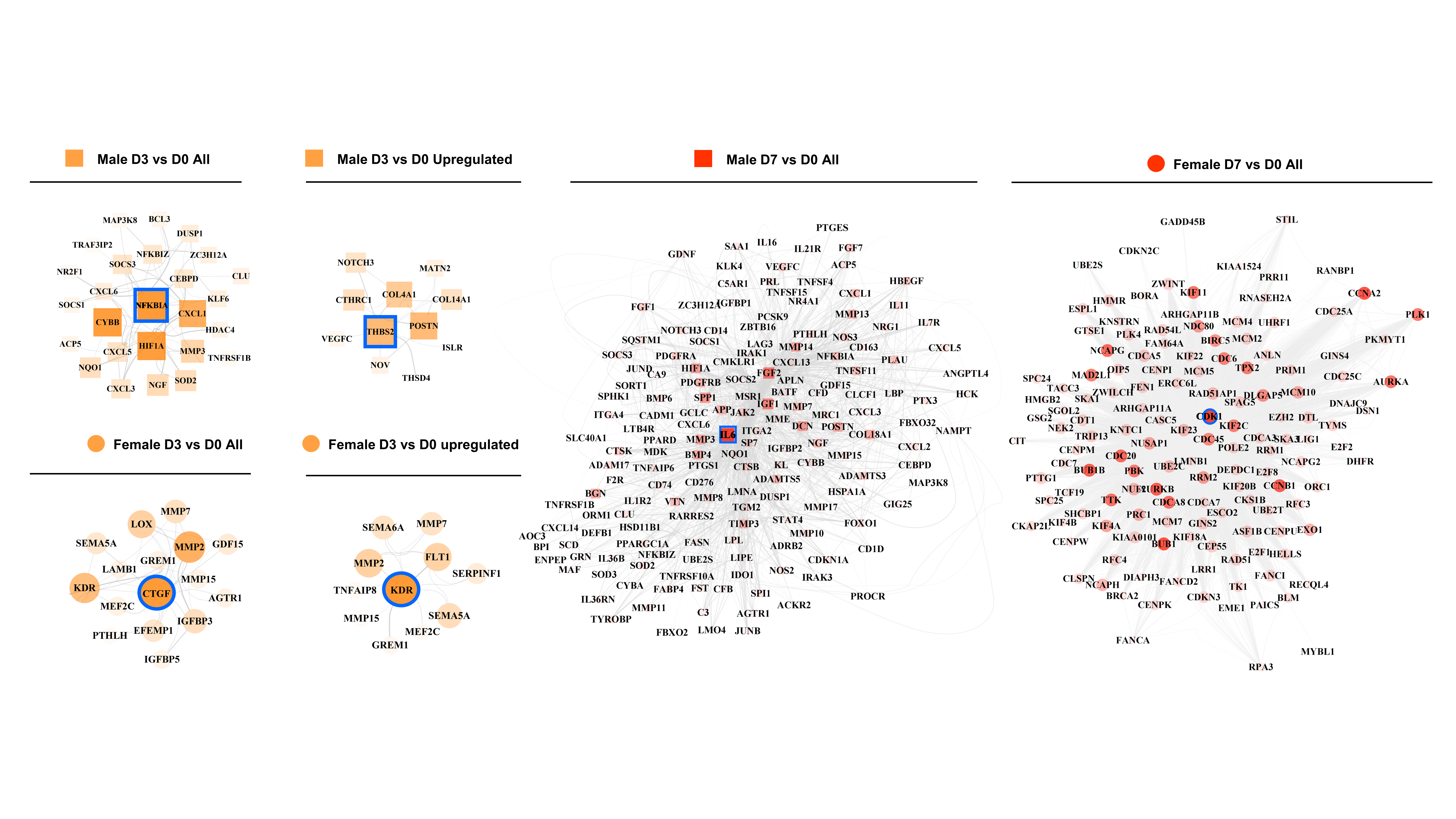

Supplement: Supplementary file 8 — Supplementary Figure 8 [file 12964_2024_1684_MOESM8_ESM.tif]
