# Supplementary material for: Short-term response of primary human meniscus cells to simulated microgravity
Source: Cell Commun Signal. 2024 Jun 21;22:342. doi: 10.1186/s12964-024-01684-w (PMC11191296; doi:10.1186/s12964-024-01684-w)
Supplement: Supplementary file 14 — Supplementary Table 6 [file 12964_2024_1684_MOESM14_ESM.docx]

| **Donor** | **Time (day)** | ***B2M*** | | ***β-Actin*** | | ***YWHAZ*** | |
| --- | --- | --- | --- | --- | --- | --- | --- |
|  |  | **Static** | **SMG** | **Static** | **SMG** | **Static** | **SMG** |
| **F1** | **0** | 20.44 | | 18.77 | | 24.08 | |
|  | **1** | 20.27 | 20.51 | 18.78 | 18.78 | 24.33 | 24.17 |
|  | **3** | 20.48 | 20.45 | 19.26 | 18.98 | 24.57 | 24.38 |
|  | **7** | 20.21 | 20.10 | 19.05 | 19.33 | 24.70 | 24.52 |
| **F2** | **0** | 21.65 | | 19.11 | | 24.97 | |
|  | **1** | 21.71 | 21.68 | 19.09 | 18.93 | 25.34 | 24.93 |
|  | **3** | 21.78 | 21.30 | 19.44 | 19.42 | 25.63 | 25.54 |
|  | **7** | 21.20 | 20.72 | 19.74 | 19.23 | 25.82 | 25.57 |
| **F3** | **0** | 21.48 | | 18.85 | | 24.91 | |
|  | **1** | 21.71 | 21.43 | 19.27 | 19.05 | 25.33 | 25.13 |
|  | **3** | 22.01 | 21.28 | 19.78 | 19.51 | 26.00 | 25.40 |
|  | **7** | 21.32 | 19.89 | 19.89 | 19.41 | 25.89 | 25.56 |
| **M1** | **0** | 19.89 | | 19.09 | | 24.93 | |
|  | **1** | 20.73 | 20.30 | 18.57 | 18.46 | 24.81 | 24.54 |
|  | **3** | 20.62 | 20.52 | 19.09 | 18.83 | 25.06 | 24.40 |
|  | **7** | 20.86 | 19.39 | 19.54 | 19.14 | 26.02 | 24.69 |
| **M2** | **0** | 19.70 | | 19.75 | | 26.79 | |
|  | **1** | 19.75 | 19.99 | 18.47 | 18.63 | 24.57 | 24.46 |
|  | **3** | 20.25 | 20.05 | 19.12 | 19.06 | 25.35 | 24.58 |
|  | **7** | 20.40 | 20.19 | 19.39 | 19.30 | 25.69 | 24.97 |
| **M3** | **0** | 19.17 | | 18.72 | | 24.59 | |
|  | **1** | 20.64 | 20.32 | 18.85 | 18.85 | 25.25 | 25.10 |
|  | **3** | 20.91 | 20.76 | 19.36 | 19.56 | 25.79 | 25.64 |
|  | **7** | 20.59 | 19.87 | 19.26 | 19.10 | 25.88 | 25.30 |

**Supplementary Table 6. CT value for the selected housekeeping genes**
